# The genomic afterlife of cancer therapy

**DOI:** 10.1172/JCI213544

**Published:** 2026-11-02

**Authors:** Xiaolu Wang, Nilay S. Sethi

**Affiliations:** 1 Department of Medical Oncology, Dana-Farber Cancer Institute, Boston, Massachusetts, USA.; 2 Department of Medicine, Harvard Medical School, Boston, Massachusetts, USA.; 3 Broad Institute of Massachusetts Institute of Technology and Harvard University, Cambridge, Massachusetts, USA.; 4 Gastrointestinal Cancer Center, Dana-Farber Cancer Institute, Boston, Massachusetts, USA.

Many environmental exposures can alter DNA sequences in recognizable ways. Tobacco smoke, ultraviolet light, aflatoxin, and colibactin each leave a characteristic pattern of DNA variants, and analyzing the pattern of this DNA variation in a cancer genome can reveal an individual’s prior exposure. Cancer therapy itself is a genotoxic exposure, delivered at recorded doses and on known dates to people we hope will live for decades. For long-term cancer survivors, the biological consequences of treatment do not end after the original cancer is cured.

In a recent report, Layeghifard and colleagues provided a broad genomic view of this pattern of DNA variants, showing that chemotherapy and radiotherapy in children with cancer can leave behind mutational signatures that are detectable after treatment, including in tumors collected at relapse (1). This work builds on a large survivorship literature showing that childhood cancer survivors carry substantial long-term morbidity and elevated risks of subsequent neoplasms (2–4). With cancer therapy’s clinical risk established and genomic tools increasingly able to read prior exposure from DNA variation, the central question is how to interpret the genetic signatures left by cancer therapy: as records of prior treatment, markers of surviving lineages, predictors of future outcomes, or clues to biology that should change survivorship care?

A somatic mutational landscape can be a record of exposure. It tells us that a cell lineage met a mutagen and then survived long enough to be sampled. In some settings, the exposure record may have a direct relationship to disease behavior: therapy generates DNA variation, a resistant clone expands, and relapse follows. In other settings, particularly decades after treatment, the signature may be better interpreted as a lineage mark or retrospective dosimeter, recording cumulative exposure to genotoxic therapies. Therapy-associated signatures therefore record exposure and lineage survival before they establish mechanism. Distinguishing what these signatures record from what they explain has become central to cancer survivorship genomics.

## Therapy written into relapsed tumors

Layeghifard et al. analyzed 611 whole-genome-sequenced tumors from 544 children across three precision-medicine programs, integrating cancer genomes with detailed treatment histories covering 86 therapeutic agents (1). This design allowed the authors to ask not only whether therapy leaves a genomic imprint, but which therapies leave one, how quickly those imprints become detectable, and whether they relate to later tumor behavior.

Tumors that progressed after therapy carried higher variant burdens than treatment-naive tumors across several classes of genomic variation, but this depended on disease stage and elapsed time (likely linked to cumulative therapy); treated primary tumors, which were sampled fewer than 4 months after therapy, did not show the same increase that was seen in treated advanced tumors (relapsed, metastatic), which were sampled nearly 2 years later. Chemotherapy and radiotherapy were the only detectable exogenous mutagens in these relapsed childhood cancers, and the effects of platinum therapy were particularly robust. Established platinum-associated signatures (i.e., SBS31, SBS35, DBS5) were enriched in treated tumors, and additional platinum-associated genomic features were identified. By integrating therapy timing with variant burden, the authors showed that platinum-associated signatures could become detectable within months, appearing as early as 91 days after therapy initiation in tumors with sufficient mutation burden. Their prevalence increased over time, reaching more than 33% of platinum-treated tumors within one year and approximately 50% by almost 3 years.

Timing is one of the most important observations of the study, because it shows that therapy signature is not a binary marker of exposure. Detection depends on dose, elapsed time, mutation burden, clonal expansion, and sampling. A tumor without a detectable therapy signature may have escaped the mutagenic process or the relevant clone may be below the limit of detection. Conversely, a signature-positive tumor does not indicate that therapy-induced mutations caused relapse; it shows that surviving cells bear the genomic record of treatment.

The resistance findings further support this interpretation. Platinum signatures were associated with poorer response to subsequent platinum treatment in independent cohorts, confirming prior clinical observations — however, this reduced response was attributed to higher expression of resistance-associated genes rather than recurrent therapy-induced driver mutations. Thus, even when therapy-induced mutagenesis is detected, the scar of exposure marks the surviving cell more than it explains the resistance mechanism.

## Therapy filters clones in normal stem cell compartments

This interpretation helps reconcile two bodies of literature that can appear to point in different directions. Layeghifard et al. describe chemotherapy as acting primarily as a mutagenic driver of clonal evolution rather than as a selective force (1). Studies in normal blood, however, suggest that, over longer timescales, therapy often functions more as a selective pressure than as a source of new drivers (5–7), favoring the expansion of rare preexisting clones, as shown by therapy-associated malignancies that share driver mutations with clones detectable before treatment (7).

These views are complementary, and their relative importance depends on compartment and timescale. In a tumor under active drug pressure, where mutations are abundant and survival is rare, therapy can create new variation in surviving cells. In a normal stem cell compartment, most mutations are lost unless the cell carrying them persists or gains a selective advantage; what therapy changes is which lineages remain. The asymmetry compounds over decades since a normal cell must still acquire every capability a tumor cell has already secured. Therapy can therefore be both mutagen and filter, and the balance changes by tissue and with time (Figure 1).

## Decades later: therapy-associated polyposis

Therapy-associated polyposis (TAP) offers a view from the opposite end of the timeline. It is a rare condition in which survivors of childhood or young adult cancers develop numerous premalignant polyps in the gastrointestinal epithelium decades after treatment, with no recognized hereditary polyposis syndrome (8, 9). Our recently published analysis in the *JCI* characterized TAP in 29 survivors of childhood or young adult cancers (10). In our cohort, patients were diagnosed with their original cancer at a median age of 16 and developed polyposis nearly three decades later. Most evaluable patients had received alkylating chemotherapy, and TAP adenomas carried SBS25, a signature linked to methyl-hydrazine alkylators such as procarbazine and dacarbazine (11), together with greater structural genomic disruption than adenomas from hereditary conditions. These were not relapsed childhood cancers but premalignant colorectal lesions arising long after cure, and their genomes extend therapy-associated signatures from relapse biology into survivorship biology.

The decades-long interval between chemotherapy exposure and development of TAP is best understood as evidence of lineage persistence. Colonic epithelium renews continuously, so a therapy-marked cell is ordinarily lost; thus, what we detect thirty years later is not preserved damage but rather a preserved lineage, one that endured treatment and eventually entered neoplastic growth (Figure 1). The SBS25 signature in TAP is therefore a record of exposure and lineage survival rather than evidence that the signature caused polyposis. TAP also illustrates why survivor genomes may carry layered exposure histories: alkylating agents leave base substitutions, whereas radiation more often imparts structural alterations.

## The missing comparator: normal tissue

A central unresolved question is whether therapy-associated signatures are specific to neoplastic cells. Layeghifard et al. primarily studied tumors, whereas the TAP adenomas in our study were premalignant. In both cases, the sampled tissue has already entered a disease trajectory involving clonal expansion. But what if the same signatures are also present to similar extents in matched nonneoplastic tissues from the same survivors? Or present in tissue of survivors without secondary neoplasms?

If they are, then finding one in a lesion is chiefly retrospective dosimetry, and the informative questions become quantitative and evolutionary. Are signature-bearing clones more abundant, more clonally expanded, more dedifferentiated, or more likely to carry driver alterations in lesions than in adjacent normal epithelium? On the other hand, if instead the signatures are rare in matched normal tissue but enriched in premalignant and malignant clones, they mark a lineage specifically involved in initiation or progression.

Some examples fall between the models used in the studies discussed above. In BRCA1/2-mutated ovarian cancer, recurrent platinum-resistant tumors can acquire secondary BRCA1/2 mutations that restore gene function, and these events were approximately 4 times more frequent in patients with a history of breast cancer (12). This finding suggests that prior therapy can shape the biology of a later malignancy by enriching for functional resistance states. Whether such reversions are induced by treatment, selected from rare preexisting cells, or both remains difficult to resolve without matched normal tissue or pretreatment clonal sampling.

## From signatures to survivorship care

Given this early understanding of genomes imprinted by prior treatment, how can we utilize them in clinical practice? Exposure reconstruction requires only that a signature be specific, and it may be especially valuable in survivorship, where treatment records can be decades old, incomplete, or difficult to retrieve. A sufficiently specific signature can provide molecular evidence that a tissue lineage experienced a particular class of therapy.

Prediction of response requires a demonstrated association with clinical outcome, which the findings related to platinum therapies begin to deliver. In patients with recurrent ovarian cancer, platinum rechallenge is guided largely by the platinum-free interval, a clinical proxy for whether the relapsing clone remains platinum-sensitive (13). A signature reflects that history directly and could eventually sharpen clinical judgement. That said, how should we interpret the presence or absence of a platinum signature in a secondary cancer of a survivor?

Risk stratification is the most challenging application for exposure reconstruction, because a signature records prior exposure more directly than it predicts future cancer. Mutation burden is not the same as cancer risk. Some therapies leave weak or undetectable base substitution signatures but still increase cancer risk. Anthracyclines leave no detectable base substitution signature in normal cells or in clonal whole-genome assays yet demonstrate a linear dose response for risk of subsequent breast cancer (14). A model in which “more signature means more future cancer” is unlikely to be correct.

Still, these studies raise a practical question. Surveillance for childhood cancer survivors emphasizes radiation field and dose, but our analyses of TAP suggest that alkylating chemotherapy may also contribute to late gastrointestinal risk. This provides a mechanistic reason to study whether a history of alkylating chemotherapy should contribute more directly to risk-adapted gastrointestinal surveillance.

The issue extends beyond the colon. Survivors of childhood cancer develop breast cancers, thyroid cancers, skin cancers, meningiomas, and other later neoplasms at higher rates than their peers (2–4); in our study, half the women with TAP developed breast cancer and more than a quarter developed basal cell carcinoma (10). In some patients, therapy-associated signatures may help reconstruct which exposure contributed to which later lesion. In others, they may reveal that normal tissues carry widespread treatment-associated scars without ever progressing to cancer. In addition to calculating secondary cancer risk, survivorship biology may ask how curative therapy reshapes normal and premalignant tissues over a lifetime.

## Reading the survivor genome correctly

The genome of a cancer survivor records more than the history of the original cancer. It may also record what we did and which cells lived through it. Layeghifard et al. showed that treatment-associated signatures can emerge within months in relapsed childhood cancers and may identify clinically relevant resistant lineages (1). Our analysis of TAP adenomas extended this idea across a much longer time scale, showing that related genomic records can be found decades later in premalignant colorectal lesions arising after cure. Between those poles lies a largely unmapped landscape of normal tissues, subsequent neoplasms, and long-latency disease.

Cancer therapy is therefore a mutagen and a selective force, acting differently across tissues and time. The task for survivorship biology is not to catalog scars, but to know when a scar is a retrospective dosimeter, when it is a lineage mark, and when it is a reason to act.

## Manuscript information

### AI disclosure.

OpenAI ChatGPT, GPT-5.5 Thinking model, was used between August and September 2026 to assist with editing manuscript text for grammar, clarity, readability, and organization. The authors reviewed and verified all AI-assisted edits and take full responsibility for the manuscript.

### Conflict of interest.

NSS serves on the scientific advisory boards of Astrin Biosciences and Debbie’s Dream Foundation; has consulted for Dewpoint Therapeutics, Bayer, and the GI Department at the First Affiliated Hospital of Sun Yat-sen University; and receives research funding from Novartis. NSS is an inventor on US patent application publication US20240280561A1 and on a separate patent application in preparation related to chromatin engineering biotechnology.

### Funding.

This Viewpoint was supported by the following:

- Department of Defense (CA201084) to NSS.
- American Cancer Society (RSG-24-1321463, DBG-25-1500423) to NSS.
- NIH/National Cancer Institute (1R01CA292507, 1R01CA325533) to NSS.
- Roxanne and Henry Brandt Foundation to NSS.

This work is the result of NIH funding, in whole or in part, and is subject to the NIH Public Access Policy. Through acceptance of this federal funding, the NIH has been given a right to make the work publicly available in PubMed Central.

## Figures and Tables

**Figure 1:**
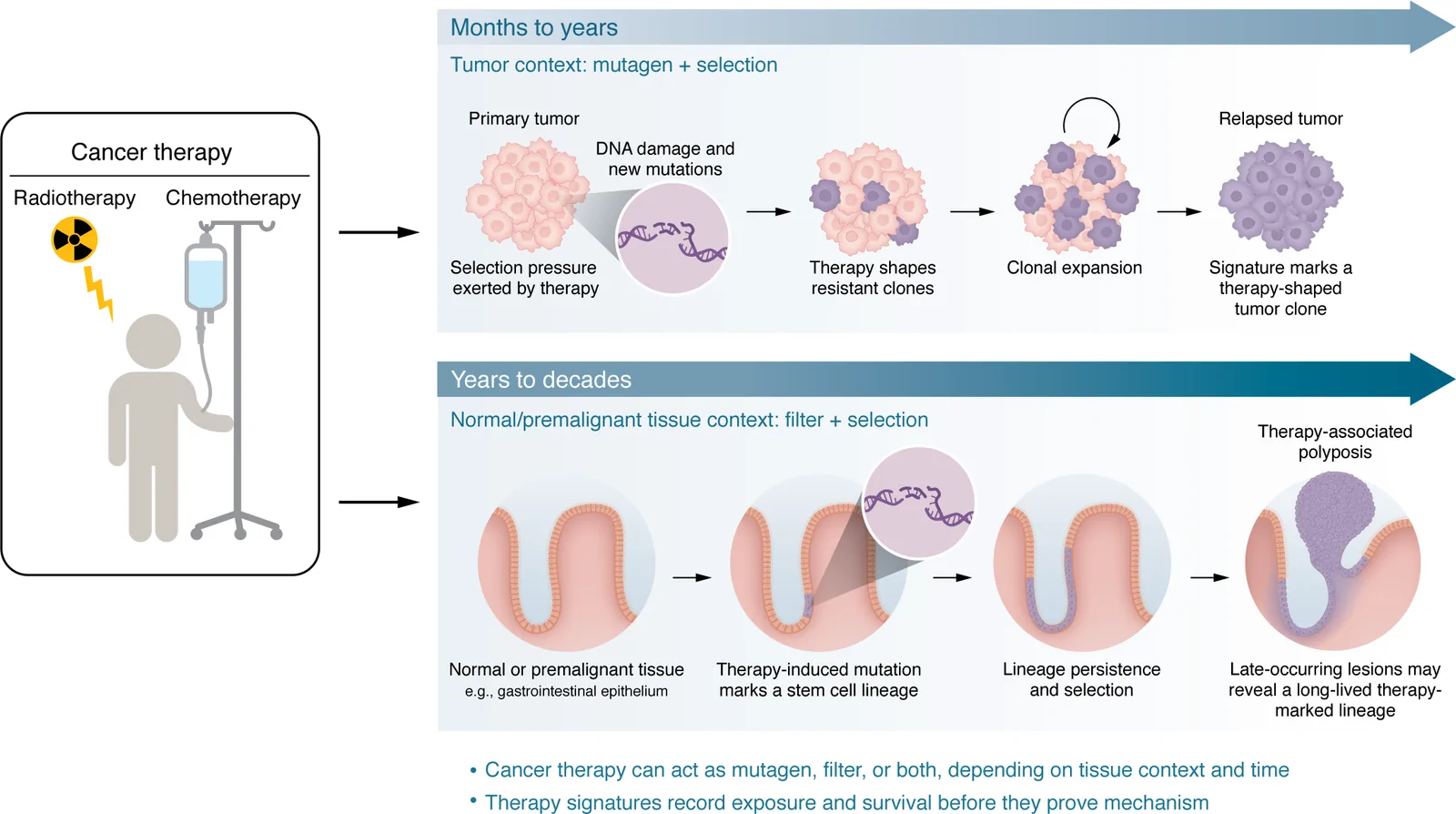
Cancer therapy leaves genomic scars and reshapes surviving cell lineages across time. Cancer therapy can introduce DNA variants while also selecting for cells that survive treatment. In relapsed tumors, therapy-associated mutational signatures can become detectable within months as surviving clones expand, as shown in ref. 1. In normal stem cell compartments, selection of surviving lineages may have consequences that emerge years or decades later. These lineages can eventually contribute to premalignant or malignant disease, as illustrated by therapy-associated polyposis (10). A therapy-associated signature therefore records not only prior treatment, but also the history of the cells that survived and expanded afterward.

## Version 1 11/02/2026

Electronic publication
